# 4-(5-Chloro-2-methyl­phen­yl)-1-[2-oxo-5-(trifluoro­meth­oxy)indolin-3-yl­idene]thio­semicarbazide

**DOI:** 10.1107/S1600536810014650

**Published:** 2010-04-24

**Authors:** Humayun Pervez, Mohammad S. Iqbal, Naveeda Saira, Muhammad Yaqub, M. Nawaz Tahir

**Affiliations:** a Department of Chemistry, Bahauddin Zakariya University, Multan 60800, Pakistan; b Department of Chemistry, Government College University, Lahore, Pakistan; c Department of Physics, University of Sargodha, Sargodha, Pakistan

## Abstract

The asymmetric unit of the title compound, C_17_H_12_ClF_3_N_4_O_2_S, contains two mol­ecules, which differ in their planarity and hydrogen bonding. In one mol­ecule, the 2-oxoindolin (C_8_/N/O *A*), thio­semicarbazide (N_3_/C/S *B*) and 5-chloro-2-methyl­phenyl (C_7_/Cl *C*) units are planar with r.m.s. deviations of 0.0110, 0.0173 and 0.0259 Å, respectively. The dihedral angles *A*/*B*, *B*/*C* and *A*/*C* are 1.74 (15), 40.70 (13) and 41.00 (11)°, respectively. In the other mol­ecule the deviations are 0.0455, 0.0007 and 0.0143 Å, respectively, and the dihedral angles are 5.01 (14), 4.53 (16) and 3.38 (13)°, respectively. In both mol­ecules, intra­molecular N—H⋯N and N—H⋯O hydrogen bonds form *S*(5) and *S*(6) ring motifs, respectively and C—H⋯S interactions occur. In one of the molecules, an intramolecular C—H⋯F interaction is also present. In the crystal, the mol­ecules are linked by N—H⋯O, C—H⋯F, C—H⋯O and N—H⋯S hydrogen bonding, forming a polymeric network.

## Related literature

For our work on 1*H*-indole-2,3-dione derivatives having pharmaceutical applications, see: Pervez *et al.* (2007 ▶, 2008 ▶, 2009*a* ▶, 2010*a* ▶). For the structures of 1-(5-nitro-2-oxoindolin-3-yl­idene)-4-*o*-tolyl­thio­semicarbazide and 4-(2-fluoro­phen­yl)-1-(2-oxoindolin-3-yl­idene)thio­semicarbazide, see: Pervez *et al.* (2009*b* ▶, 2010*b* ▶). For hydrogen-bond motifs, see: Bernstein *et al.* (1995 ▶).
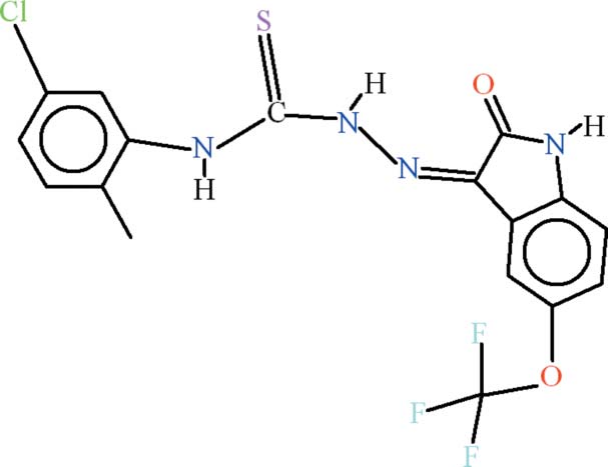


## Experimental

### 

#### Crystal data

C_17_H_12_ClF_3_N_4_O_2_S
*M* *_r_* = 428.82
Triclinic, 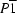

*a* = 10.5808 (5) Å
*b* = 11.0262 (5) Å
*c* = 17.1743 (7) Å
α = 102.855 (3)°
β = 94.766 (3)°
γ = 107.814 (2)°
*V* = 1834.96 (15) Å^3^
*Z* = 4
Mo *K*α radiation
μ = 0.37 mm^−1^
*T* = 296 K
0.24 × 0.18 × 0.16 mm

#### Data collection

Bruker Kappa APEXII CCD diffractometer
Absorption correction: multi-scan (*SADABS*; Bruker, 2005 ▶) *T*_min_ = 0.924, *T*_max_ = 0.945
33611 measured reflections
6822 independent reflections
3927 reflections with *I* > 2σ(*I*)
*R*_int_ = 0.049

#### Refinement

*R*[*F* ^2^ > 2σ(*F* ^2^)] = 0.051
*wR*(*F* ^2^) = 0.145
*S* = 1.02
6822 reflections
507 parameters
H-atom parameters constrained
Δρ_max_ = 0.35 e Å^−3^
Δρ_min_ = −0.36 e Å^−3^

### 

Data collection: *APEX2* (Bruker, 2007 ▶); cell refinement: *SAINT* (Bruker, 2007 ▶); data reduction: *SAINT*; program(s) used to solve structure: *SHELXS97* (Sheldrick, 2008 ▶); program(s) used to refine structure: *SHELXL97* (Sheldrick, 2008 ▶); molecular graphics: *ORTEP-3 for Windows* (Farrugia, 1997 ▶) and *PLATON* (Spek, 2009 ▶); software used to prepare material for publication: *WinGX* (Farrugia, 1999 ▶) and *PLATON*.

## Supplementary Material

Crystal structure: contains datablocks global, I. DOI: 10.1107/S1600536810014650/bq2207sup1.cif

Structure factors: contains datablocks I. DOI: 10.1107/S1600536810014650/bq2207Isup2.hkl

Additional supplementary materials:  crystallographic information; 3D view; checkCIF report

## Figures and Tables

**Table 1:** Hydrogen-bond geometry (Å, °)

*D*—H⋯*A*	*D*—H	H⋯*A*	*D*⋯*A*	*D*—H⋯*A*
N1—H1⋯O3^i^	0.8600	2.1800	2.937 (3)	147.00
N3—H3⋯O1	0.8600	2.2100	2.841 (3)	131.00
N4—H4*A*⋯N2	0.8600	2.0900	2.553 (4)	113.00
N5—H5⋯S1^ii^	0.8600	2.6400	3.287 (3)	133.00
N7—H7*A*⋯O3	0.8600	2.2200	2.863 (3)	132.00
N8—H8⋯N6	0.8600	2.0600	2.546 (3)	115.00
C12—H12⋯F6^iii^	0.9300	2.4700	3.284 (6)	147.00
C15—H15⋯S1	0.9300	2.8700	3.325 (3)	111.00
C21—H21⋯F5	0.9300	2.3800	2.930 (5)	118.00
C32—H32⋯S2	0.9300	2.5700	3.245 (4)	130.00
C33—H33*C*⋯O1	0.9600	2.4000	3.355 (4)	176.00

Symmetry codes: (i) 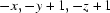; (ii) 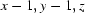; (iii) 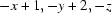.

**Fig. 1.**
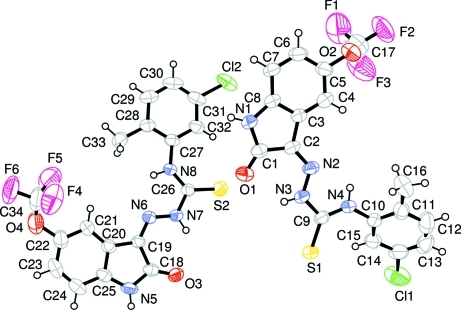
View of (I) with the atom numbering scheme. The thermal ellipsoids are drawn at the 50% probability level.

**Fig. 2.**
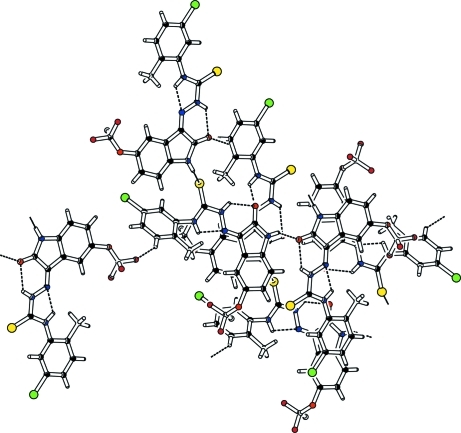
The partial packing of (I) (PLATON; Spek, 2009) which shows that molecules form ring motifs and form polymeric network.

## supplementary crystallographic information

### Comment

In continuation of our work on 1*H*-indole-2,3-dione derivatives having pharmaceutical applications (Pervez *et al.*, 2007, 2008, 2009a, 2010a), we report herein the synthesis and crystal structure of the title compound (I, Fig. 1).

The crystal structures of (II) *i.e.* 1-(5-nitro-2-oxoindolin-3-ylidene)-4-*o*-tolylthiosemicarbazide (Pervez *et al.*, 2009b) and (III) *i.e.* 4-(2-fluorophenyl)-1-(2-oxoindolin-3-ylidene)thiosemicarbazide (Pervez *et al.*, 2010b) have been published previously. The title compound (I) differs from (II) due to the presence of trifluoromethoxy instead of nitro function and an additional chloro group at position-5 of the isatin scaffold and phenyl ring substituted at N^4^ of the thiosemicarbazone moiety, respectively. From (III), it differs due to the presence of trifluoromethoxy group at position-5 of the isatin scaffold, and methyl instead of fluoro and an additional chloro function at position-5 of the phenyl ring substituted at N^4^ of the thiosemicarbazone moiety.

The asymmetric unit of title compound consist of two molecules. In one molecule, the 2-oxoindolin A (C1–C8/N1/O1), thiosemicarbazide B (N2/N3/C9/S1/N4) and the 5-Chloro-2-methylphenyl C (C10–C16/Cl1) are planar with maximum r.m.s. deviations of 0.0110 Å, 0.0173 Å and 0.0259 Å, respectively. The dihedral angles between A/B, B/C and A/C are 1.74 (15)°, 40.70 (13)° and 41.00 (11)°, respectively. In the other molecule the same groups D (C18—C25/N5/O3), E (N6/N7/C26/S2/N8) and F (C27—C33/CL2) are also planar with maximum r.m.s. deviations of 0.0455 Å, 0.0007 Å and 0.0143 Å, respectively. The dihedral angles between D/E, E/F and D/F are 5.01 (14)°, 4.53 (16)° and 3.38 (13)°, respectively. These values show that the later molecule is more planar than the former. Due to intramolecular H-bondings (Table 1, Fig. 2), S(5) and S(6) (Bernstein *et al.*, 1995) ring motifs are formed in each molecule. The molecules are stabilized in the form of polymeric network through intermolecular H-bonding of types N—H···O , C—H···F and N—H···S.

### Experimental

4-(5-Chloro-2-methylphenyl)thiosemicarbazide (0.86 g, 4.0 mmol) dissolved in ethanol (10 ml) was added to a hot solution of 5-(trifluoromethoxy) indolin-2,3-dione (0.92 g, 4.0 mmol) in 50% aqueous ethanol (20 ml) containing a catalytic quantity of glacial acetic acid. The reaction mixture was then refluxed for 2 h. The orange colored amorphous solid formed during refluxing was collected by suction filtration. Thorough washing with hot aqueous ethanol afforded the title compound (I) in pure form (1.10 g, 64%), m.p. 513 K. The crystals of (I) for x-ray analysis were grown in ethyl acetate-petroleum ether (2:5) solvent system at room temperature by diffusion method.

### Refinement

The H-atoms were positioned geometrically (C–H = 0.93–0.96 Å, C–N = 0.86 Å) and refined as riding with *U*_iso_(H) = x*U*_eq_(C, N), where x = 1.5 for methyl and 1.2 for other H-atoms.

### Crystal data

**Table:**

C_17_H_12_ClF_3_N_4_O_2_S	*Z* = 4
*M**_r_* = 428.82	*F*(000) = 872
Triclinic, *P*1	*D*_x_ = 1.552 Mg m^−^^3^
Hall symbol: -P 1	Mo *K*α radiation, λ = 0.71073 Å
*a* = 10.5808 (5) Å	Cell parameters from 6822 reflections
*b* = 11.0262 (5) Å	θ = 2.1–25.5°
*c* = 17.1743 (7) Å	µ = 0.37 mm^−^^1^
α = 102.855 (3)°	*T* = 296 K
β = 94.766 (3)°	Prism, red
γ = 107.814 (2)°	0.24 × 0.18 × 0.16 mm
*V* = 1834.96 (15) Å^3^	

### Data collection

**Table:**

Bruker Kappa APEXII CCD diffractometer	6822 independent reflections
Radiation source: fine-focus sealed tube	3927 reflections with *I* > 2σ(*I*)
graphite	*R*_int_ = 0.049
Detector resolution: 7.80 pixels mm^-1^	θ_max_ = 25.5°, θ_min_ = 2.1°
ω scans	*h* = −12→12
Absorption correction: multi-scan (*SADABS*; Bruker, 2005)	*k* = −13→13
*T*_min_ = 0.924, *T*_max_ = 0.945	*l* = −20→20
33611 measured reflections	

### Refinement

**Table:**

Refinement on *F*^2^	Primary atom site location: structure-invariant direct methods
Least-squares matrix: full	Secondary atom site location: difference Fourier map
*R*[*F*^2^ > 2σ(*F*^2^)] = 0.051	Hydrogen site location: inferred from neighbouring sites
*wR*(*F*^2^) = 0.145	H-atom parameters constrained
*S* = 1.02	*w* = 1/[σ^2^(*F*_o_^2^) + (0.0588*P*)^2^ + 0.8346*P*] where *P* = (*F*_o_^2^ + 2*F*_c_^2^)/3
6822 reflections	(Δ/σ)_max_ < 0.001
507 parameters	Δρ_max_ = 0.35 e Å^−^^3^
0 restraints	Δρ_min_ = −0.36 e Å^−^^3^

### Special details

**Table:**

Geometry. Bond distances, angles etc. have been calculated using the rounded fractional coordinates. All su's are estimated from the variances of the (full) variance-covariance matrix. The cell esds are taken into account in the estimation of distances, angles and torsion angles
Refinement. Refinement of *F*^2^ against ALL reflections. The weighted *R*-factor *wR* and goodness of fit *S* are based on *F*^2^, conventional *R*-factors *R* are based on *F*, with *F* set to zero for negative *F*^2^. The threshold expression of *F*^2^ > σ(*F*^2^) is used only for calculating *R*-factors(gt) *etc*. and is not relevant to the choice of reflections for refinement. *R*-factors based on *F*^2^ are statistically about twice as large as those based on *F*, and *R*- factors based on ALL data will be even larger.

### Fractional atomic coordinates and isotropic or equivalent isotropic displacement parameters (Å^2^)

**Table:**

	*x*	*y*	*z*	*U*_iso_*/*U*_eq_	
Cl1	0.90141 (12)	1.08034 (18)	0.04378 (8)	0.1311 (7)	
S1	0.73013 (8)	1.08876 (10)	0.33045 (6)	0.0663 (4)	
F1	−0.2587 (4)	0.5431 (3)	0.0906 (2)	0.1816 (18)	
F2	−0.2738 (3)	0.6739 (3)	0.02089 (18)	0.1430 (16)	
F3	−0.0876 (4)	0.6495 (4)	0.05415 (19)	0.167 (2)	
O1	0.3721 (2)	0.8851 (2)	0.46034 (14)	0.0616 (9)	
O2	−0.1786 (3)	0.7545 (3)	0.14412 (19)	0.0825 (11)	
N1	0.1403 (2)	0.7922 (2)	0.42522 (16)	0.0498 (9)	
N2	0.3438 (3)	0.9308 (2)	0.29315 (17)	0.0503 (9)	
N3	0.4732 (2)	0.9813 (2)	0.32968 (17)	0.0522 (9)	
N4	0.5207 (3)	1.0337 (3)	0.21286 (17)	0.0585 (10)	
C1	0.2674 (3)	0.8546 (3)	0.4138 (2)	0.0494 (11)	
C2	0.2519 (3)	0.8751 (3)	0.3308 (2)	0.0472 (11)	
C3	0.1102 (3)	0.8184 (3)	0.29786 (19)	0.0455 (11)	
C4	0.0382 (3)	0.8115 (3)	0.2255 (2)	0.0552 (12)	
C5	−0.1000 (3)	0.7532 (3)	0.2146 (2)	0.0606 (14)	
C6	−0.1641 (3)	0.7044 (3)	0.2728 (2)	0.0646 (14)	
C7	−0.0922 (3)	0.7113 (3)	0.3450 (2)	0.0587 (14)	
C8	0.0459 (3)	0.7697 (3)	0.35726 (19)	0.0457 (11)	
C9	0.5704 (3)	1.0342 (3)	0.2870 (2)	0.0504 (11)	
C10	0.5856 (3)	1.0824 (3)	0.1519 (2)	0.0552 (12)	
C11	0.5210 (4)	1.1454 (3)	0.1090 (2)	0.0644 (14)	
C12	0.5790 (5)	1.1884 (4)	0.0471 (3)	0.0876 (19)	
C13	0.6963 (5)	1.1717 (5)	0.0280 (3)	0.0936 (19)	
C14	0.7571 (4)	1.1069 (4)	0.0704 (2)	0.0796 (16)	
C15	0.7029 (3)	1.0613 (4)	0.1325 (2)	0.0666 (14)	
C16	0.3887 (4)	1.1624 (4)	0.1257 (2)	0.0859 (17)	
C17	−0.1989 (6)	0.6580 (7)	0.0794 (4)	0.107 (3)	
Cl2	0.91864 (10)	0.86095 (10)	0.77449 (6)	0.0815 (4)	
S2	0.40866 (10)	0.58900 (11)	0.67685 (6)	0.0801 (4)	
F4	0.4264 (4)	0.5047 (4)	0.1554 (2)	0.1422 (17)	
F5	0.4754 (3)	0.7019 (3)	0.2115 (2)	0.1348 (14)	
F6	0.4451 (3)	0.6393 (4)	0.08329 (19)	0.1460 (16)	
O3	0.0157 (2)	0.3691 (2)	0.47803 (14)	0.0558 (8)	
O4	0.2739 (4)	0.5967 (4)	0.1420 (2)	0.1177 (16)	
N5	−0.0455 (3)	0.3792 (3)	0.34712 (17)	0.0574 (10)	
N6	0.2839 (2)	0.5485 (2)	0.45305 (15)	0.0460 (9)	
N7	0.2893 (2)	0.5392 (2)	0.52977 (15)	0.0482 (9)	
N8	0.5052 (2)	0.6698 (3)	0.54981 (16)	0.0540 (9)	
C18	0.0396 (3)	0.4055 (3)	0.4167 (2)	0.0470 (11)	
C19	0.1729 (3)	0.4892 (3)	0.40341 (18)	0.0431 (10)	
C20	0.1555 (3)	0.4956 (3)	0.32001 (19)	0.0477 (11)	
C21	0.2453 (3)	0.5523 (3)	0.2743 (2)	0.0610 (12)	
C22	0.1959 (4)	0.5391 (4)	0.1956 (2)	0.0720 (16)	
C23	0.0625 (5)	0.4747 (4)	0.1631 (2)	0.0767 (16)	
C24	−0.0288 (4)	0.4190 (4)	0.2091 (2)	0.0694 (12)	
C25	0.0206 (3)	0.4288 (3)	0.2880 (2)	0.0530 (11)	
C26	0.4070 (3)	0.6034 (3)	0.58372 (18)	0.0458 (10)	
C27	0.6420 (3)	0.7470 (3)	0.5784 (2)	0.0466 (10)	
C28	0.7111 (3)	0.8068 (3)	0.5231 (2)	0.0475 (11)	
C29	0.8460 (3)	0.8802 (3)	0.5489 (3)	0.0613 (14)	
C30	0.9113 (3)	0.8970 (3)	0.6241 (3)	0.0653 (14)	
C31	0.8411 (3)	0.8388 (3)	0.6771 (2)	0.0588 (14)	
C32	0.7060 (3)	0.7626 (3)	0.6551 (2)	0.0545 (11)	
C33	0.6436 (3)	0.7917 (3)	0.4397 (2)	0.0634 (14)	
C34	0.3975 (7)	0.6097 (6)	0.1467 (3)	0.104 (2)	
H1	0.12071	0.76958	0.46871	0.0597*	
H3	0.49461	0.98045	0.37892	0.0626*	
H4	0.08074	0.84453	0.18566	0.0664*	
H4A	0.43479	0.99779	0.20002	0.0702*	
H6	−0.25734	0.66626	0.26341	0.0777*	
H7	−0.13535	0.67770	0.38442	0.0699*	
H12	0.53705	1.23023	0.01730	0.1050*	
H13	0.73471	1.20383	−0.01326	0.1123*	
H15	0.74409	1.01713	0.16095	0.0798*	
H16A	0.36321	1.21142	0.09122	0.1291*	
H16B	0.39812	1.20921	0.18129	0.1291*	
H16C	0.32068	1.07722	0.11524	0.1291*	
H5	−0.13038	0.33675	0.34011	0.0688*	
H7A	0.21924	0.49298	0.54511	0.0579*	
H8	0.47995	0.66451	0.49991	0.0648*	
H21	0.33551	0.59747	0.29581	0.0737*	
H23	0.03314	0.46849	0.10931	0.0919*	
H24	−0.11948	0.37683	0.18770	0.0829*	
H29	0.89418	0.91994	0.51308	0.0734*	
H30	1.00208	0.94708	0.63931	0.0786*	
H32	0.65949	0.72279	0.69143	0.0653*	
H33A	0.70436	0.84749	0.41352	0.0949*	
H33B	0.61878	0.70148	0.40892	0.0949*	
H33C	0.56448	0.81664	0.44308	0.0949*	

### Atomic displacement parameters (Å^2^)

**Table:**

	*U*^11^	*U*^22^	*U*^33^	*U*^12^	*U*^13^	*U*^23^
Cl1	0.0695 (8)	0.2284 (17)	0.0752 (8)	0.0309 (9)	0.0249 (6)	0.0215 (9)
S1	0.0366 (5)	0.0842 (7)	0.0749 (7)	0.0045 (4)	0.0044 (4)	0.0380 (5)
F1	0.236 (4)	0.099 (2)	0.140 (3)	−0.011 (3)	−0.058 (3)	0.019 (2)
F2	0.142 (3)	0.175 (3)	0.095 (2)	0.045 (2)	−0.042 (2)	0.035 (2)
F3	0.154 (3)	0.267 (5)	0.086 (2)	0.105 (3)	0.011 (2)	0.010 (2)
O1	0.0414 (13)	0.0727 (16)	0.0665 (16)	0.0138 (12)	0.0039 (12)	0.0189 (12)
O2	0.0667 (18)	0.093 (2)	0.082 (2)	0.0219 (15)	−0.0078 (16)	0.0253 (17)
N1	0.0394 (15)	0.0537 (16)	0.0560 (18)	0.0107 (12)	0.0131 (13)	0.0189 (13)
N2	0.0359 (15)	0.0474 (15)	0.0676 (18)	0.0107 (12)	0.0108 (13)	0.0187 (14)
N3	0.0374 (15)	0.0556 (16)	0.0633 (18)	0.0077 (12)	0.0101 (13)	0.0254 (14)
N4	0.0346 (14)	0.0734 (19)	0.0626 (19)	0.0031 (13)	0.0038 (14)	0.0303 (15)
C1	0.044 (2)	0.0438 (18)	0.061 (2)	0.0150 (15)	0.0143 (17)	0.0126 (16)
C2	0.0379 (18)	0.0396 (17)	0.064 (2)	0.0102 (14)	0.0147 (16)	0.0149 (15)
C3	0.0398 (18)	0.0387 (17)	0.056 (2)	0.0107 (14)	0.0100 (16)	0.0113 (15)
C4	0.052 (2)	0.051 (2)	0.064 (2)	0.0149 (16)	0.0162 (18)	0.0184 (17)
C5	0.045 (2)	0.062 (2)	0.071 (3)	0.0138 (17)	−0.0010 (18)	0.0194 (19)
C6	0.0373 (19)	0.069 (2)	0.084 (3)	0.0099 (17)	0.0101 (19)	0.024 (2)
C7	0.0380 (19)	0.064 (2)	0.074 (3)	0.0087 (16)	0.0152 (18)	0.0275 (19)
C8	0.0402 (18)	0.0387 (17)	0.058 (2)	0.0120 (14)	0.0141 (16)	0.0119 (15)
C9	0.0404 (18)	0.0454 (18)	0.065 (2)	0.0081 (15)	0.0110 (16)	0.0216 (16)
C10	0.050 (2)	0.057 (2)	0.047 (2)	0.0020 (16)	−0.0002 (16)	0.0159 (17)
C11	0.078 (3)	0.057 (2)	0.047 (2)	0.0118 (19)	−0.0008 (19)	0.0103 (17)
C12	0.118 (4)	0.088 (3)	0.058 (3)	0.030 (3)	0.011 (3)	0.029 (2)
C13	0.108 (4)	0.097 (3)	0.053 (3)	−0.003 (3)	0.015 (3)	0.026 (2)
C14	0.058 (2)	0.099 (3)	0.056 (3)	−0.002 (2)	0.009 (2)	0.009 (2)
C15	0.050 (2)	0.084 (3)	0.058 (2)	0.0109 (19)	0.0067 (18)	0.020 (2)
C16	0.098 (3)	0.104 (3)	0.066 (3)	0.055 (3)	−0.001 (2)	0.018 (2)
C17	0.087 (4)	0.135 (5)	0.085 (4)	0.023 (4)	−0.011 (3)	0.033 (4)
Cl2	0.0671 (6)	0.0745 (7)	0.0856 (7)	0.0259 (5)	−0.0283 (5)	−0.0016 (5)
S2	0.0708 (7)	0.0896 (7)	0.0513 (6)	−0.0154 (5)	−0.0063 (5)	0.0270 (5)
F4	0.186 (3)	0.154 (3)	0.144 (3)	0.096 (3)	0.083 (2)	0.077 (2)
F5	0.138 (3)	0.127 (2)	0.110 (2)	0.001 (2)	0.033 (2)	0.029 (2)
F6	0.164 (3)	0.208 (3)	0.106 (2)	0.065 (3)	0.065 (2)	0.101 (2)
O3	0.0511 (13)	0.0498 (13)	0.0574 (15)	0.0047 (10)	0.0092 (11)	0.0128 (11)
O4	0.094 (2)	0.195 (4)	0.086 (2)	0.048 (3)	0.019 (2)	0.079 (2)
N5	0.0376 (15)	0.0573 (17)	0.0593 (19)	0.0010 (13)	−0.0052 (14)	0.0053 (14)
N6	0.0423 (15)	0.0445 (15)	0.0426 (16)	0.0062 (12)	0.0007 (12)	0.0082 (12)
N7	0.0407 (15)	0.0456 (15)	0.0450 (16)	−0.0020 (12)	0.0001 (12)	0.0108 (12)
N8	0.0378 (15)	0.0667 (18)	0.0464 (16)	0.0001 (13)	−0.0026 (12)	0.0205 (14)
C18	0.0428 (19)	0.0360 (17)	0.054 (2)	0.0095 (14)	0.0022 (16)	0.0027 (15)
C19	0.0413 (18)	0.0376 (16)	0.0441 (19)	0.0080 (14)	0.0017 (14)	0.0072 (14)
C20	0.0481 (19)	0.0413 (18)	0.048 (2)	0.0113 (15)	0.0013 (16)	0.0076 (14)
C21	0.055 (2)	0.071 (2)	0.053 (2)	0.0143 (18)	−0.0011 (18)	0.0215 (18)
C22	0.077 (3)	0.090 (3)	0.056 (2)	0.030 (2)	0.011 (2)	0.030 (2)
C23	0.093 (3)	0.091 (3)	0.047 (2)	0.036 (3)	−0.007 (2)	0.019 (2)
C24	0.066 (2)	0.069 (2)	0.058 (2)	0.019 (2)	−0.015 (2)	0.0007 (19)
C25	0.051 (2)	0.0454 (19)	0.050 (2)	0.0111 (16)	−0.0055 (17)	−0.0003 (16)
C26	0.0430 (18)	0.0397 (17)	0.0457 (19)	0.0050 (14)	−0.0019 (15)	0.0088 (14)
C27	0.0359 (17)	0.0374 (17)	0.061 (2)	0.0091 (14)	−0.0010 (16)	0.0095 (15)
C28	0.0397 (18)	0.0409 (17)	0.063 (2)	0.0167 (14)	0.0095 (16)	0.0111 (15)
C29	0.041 (2)	0.049 (2)	0.092 (3)	0.0141 (16)	0.015 (2)	0.0147 (19)
C30	0.0362 (19)	0.051 (2)	0.097 (3)	0.0117 (16)	0.003 (2)	0.003 (2)
C31	0.046 (2)	0.0453 (19)	0.075 (3)	0.0206 (17)	−0.0128 (18)	−0.0033 (17)
C32	0.047 (2)	0.0478 (19)	0.060 (2)	0.0107 (16)	−0.0034 (17)	0.0084 (16)
C33	0.052 (2)	0.072 (2)	0.075 (3)	0.0215 (18)	0.0204 (19)	0.032 (2)
C34	0.146 (5)	0.105 (4)	0.064 (3)	0.029 (4)	0.026 (4)	0.044 (3)

### Geometric parameters (Å, °)

**Table:**

Cl1—C14	1.719 (5)	C6—C7	1.374 (5)
Cl2—C31	1.732 (3)	C7—C8	1.381 (5)
S1—C9	1.656 (3)	C10—C15	1.384 (5)
S2—C26	1.641 (3)	C10—C11	1.388 (5)
F1—C17	1.296 (8)	C11—C12	1.373 (6)
F2—C17	1.304 (7)	C11—C16	1.510 (6)
F3—C17	1.311 (8)	C12—C13	1.365 (8)
F4—C34	1.320 (8)	C13—C14	1.375 (7)
F5—C34	1.335 (7)	C14—C15	1.372 (5)
F6—C34	1.302 (7)	C4—H4	0.9300
O1—C1	1.218 (4)	C6—H6	0.9300
O2—C5	1.416 (5)	C7—H7	0.9300
O2—C17	1.307 (8)	C12—H12	0.9300
O3—C18	1.227 (4)	C13—H13	0.9300
O4—C22	1.413 (6)	C15—H15	0.9300
O4—C34	1.265 (9)	C16—H16A	0.9600
N1—C8	1.396 (4)	C16—H16C	0.9600
N1—C1	1.363 (4)	C16—H16B	0.9600
N2—N3	1.347 (4)	C18—C19	1.498 (5)
N2—C2	1.287 (4)	C19—C20	1.449 (4)
N3—C9	1.373 (4)	C20—C21	1.374 (5)
N4—C9	1.335 (4)	C20—C25	1.389 (5)
N4—C10	1.414 (4)	C21—C22	1.367 (5)
N1—H1	0.8600	C22—C23	1.374 (7)
N3—H3	0.8600	C23—C24	1.384 (6)
N4—H4A	0.8600	C24—C25	1.380 (5)
N5—C25	1.397 (5)	C27—C28	1.404 (5)
N5—C18	1.353 (4)	C27—C32	1.380 (5)
N6—C19	1.284 (4)	C28—C33	1.497 (5)
N6—N7	1.342 (3)	C28—C29	1.385 (5)
N7—C26	1.374 (4)	C29—C30	1.359 (7)
N8—C27	1.415 (4)	C30—C31	1.369 (5)
N8—C26	1.334 (4)	C31—C32	1.388 (5)
N5—H5	0.8600	C21—H21	0.9300
N7—H7A	0.8600	C23—H23	0.9300
N8—H8	0.8600	C24—H24	0.9300
C1—C2	1.496 (5)	C29—H29	0.9300
C2—C3	1.445 (5)	C30—H30	0.9300
C3—C8	1.388 (5)	C32—H32	0.9300
C3—C4	1.377 (5)	C33—H33A	0.9600
C4—C5	1.383 (5)	C33—H33B	0.9600
C5—C6	1.370 (5)	C33—H33C	0.9600
			
Cl1···Cl1^i^	3.369 (2)	C21···F4	2.984 (5)
Cl1···Cl2^ii^	3.3384 (17)	C21···C3	3.599 (5)
Cl2···C25^iii^	3.409 (3)	C21···C2	3.452 (5)
Cl2···C4^iv^	3.499 (3)	C21···F5	2.930 (5)
Cl2···Cl1^ii^	3.3384 (17)	C21···C1	3.575 (5)
S1···N5^v^	3.287 (3)	C22···C3	3.600 (5)
S1···C15	3.325 (3)	C25···Cl2^iii^	3.409 (3)
S2···C32	3.245 (4)	C25···C8	3.600 (5)
S1···H15	2.8700	C28···C1^iv^	3.578 (5)
S1···H30^ii^	3.0000	C29···C2^iv^	3.494 (5)
S1···H5^v^	2.6400	C29···C1^iv^	3.441 (5)
S2···H33B^iii^	3.1300	C30···O3^iii^	3.406 (4)
S2···H32	2.5700	C30···C2^iv^	3.451 (5)
S2···H16B^iv^	3.0100	C30···C4^iv^	3.509 (5)
F1···C6	3.142 (5)	C30···C18^iii^	3.459 (5)
F3···C4	3.030 (5)	C30···C8^iv^	3.500 (5)
F4···C21	2.984 (5)	C30···C3^iv^	3.211 (5)
F5···C21	2.930 (5)	C31···C3^iv^	3.576 (5)
F6···C12^vi^	3.284 (6)	C31···C19^iii^	3.525 (5)
F2···H16A^vii^	2.7800	C31···C18^iii^	3.454 (5)
F3···H4	2.7800	C32···C19^iii^	3.393 (5)
F4···H21	2.7700	C32···S2	3.245 (4)
F5···H21	2.3800	C33···O1	3.355 (4)
F6···H12^vi^	2.4700	C33···C7^xi^	3.588 (5)
O1···C33	3.355 (4)	C1···H3	2.5800
O1···N3	2.841 (3)	C9···H15	2.9600
O1···N2	3.030 (4)	C14···H24^v^	3.0200
O1···O1^iv^	3.043 (3)	C16···H4A	2.5700
O3···N6	3.049 (3)	C18···H7A	2.5800
O3···N1^viii^	2.937 (3)	C26···H32	2.8700
O3···N7	2.863 (3)	C32···H16B^iv^	3.0900
O3···O3^viii^	2.945 (3)	C33···H7^xi^	3.1000
O3···C18^viii^	2.976 (4)	C33···H8	2.3400
O3···C30^iii^	3.406 (4)	C34···H21	2.7200
O1···H3	2.2100	H1···O3^viii^	2.1800
O1···H33C	2.4000	H3···H33C	2.5700
O1···H3^iv^	2.8400	H3···C1	2.5800
O3···H7A	2.2200	H3···O1	2.2100
O3···H7^viii^	2.8100	H3···O1^iv^	2.8400
O3···H1^viii^	2.1800	H4···F3	2.7800
N1···O3^viii^	2.937 (3)	H4A···N2	2.0900
N1···C20	3.424 (4)	H4A···C16	2.5700
N1···C19	3.405 (4)	H4A···H16B	2.5600
N2···N4	2.553 (4)	H4A···H16C	2.3000
N2···O1	3.030 (4)	H5···S1^ix^	2.6400
N3···O1	2.841 (3)	H7···C33^x^	3.1000
N4···N2	2.553 (4)	H7···O3^viii^	2.8100
N5···S1^ix^	3.287 (3)	H7···H7A^viii^	2.4400
N6···N8	2.546 (3)	H7A···H7^viii^	2.4400
N6···O3	3.049 (3)	H7A···O3	2.2200
N7···O3	2.863 (3)	H7A···C18	2.5800
N8···N6	2.546 (3)	H8···N6	2.0600
N2···H4A	2.0900	H8···H33C	2.1300
N4···H16B	2.7600	H8···C33	2.3400
N4···H16C	2.8100	H8···H33B	2.2500
N6···H21	2.9300	H12···H16A	2.3000
N6···H8	2.0600	H12···F6^vi^	2.4700
N8···H33B	2.8300	H15···S1	2.8700
N8···H33C	2.7000	H15···C9	2.9600
C1···C21	3.575 (5)	H16A···H12	2.3000
C1···C29^iv^	3.441 (5)	H16A···F2^vii^	2.7800
C1···C28^iv^	3.578 (5)	H16B···N4	2.7600
C2···C21	3.452 (5)	H16B···H4A	2.5600
C2···C30^iv^	3.451 (5)	H16B···S2^iv^	3.0100
C2···C29^iv^	3.494 (5)	H16B···C32^iv^	3.0900
C3···C22	3.600 (5)	H16B···H32^iv^	2.3400
C3···C21	3.599 (5)	H16C···N4	2.8100
C3···C31^iv^	3.576 (5)	H16C···H4A	2.3000
C3···C30^iv^	3.211 (5)	H21···F4	2.7700
C4···F3	3.030 (5)	H21···F5	2.3800
C4···C30^iv^	3.509 (5)	H21···N6	2.9300
C4···Cl2^iv^	3.499 (3)	H21···C34	2.7200
C6···F1	3.142 (5)	H24···C14^ix^	3.0200
C7···C33^x^	3.588 (5)	H29···H33A	2.3300
C8···C30^iv^	3.500 (5)	H29···H29^ii^	2.5400
C8···C20	3.507 (5)	H30···S1^ii^	3.0000
C8···C25	3.600 (5)	H32···S2	2.5700
C12···F6^vi^	3.284 (6)	H32···C26	2.8700
C15···S1	3.325 (3)	H32···H16B^iv^	2.3400
C18···C31^iii^	3.454 (5)	H33A···H29	2.3300
C18···C30^iii^	3.459 (5)	H33B···N8	2.8300
C18···O3^viii^	2.976 (4)	H33B···H8	2.2500
C18···C18^viii^	3.471 (5)	H33B···S2^iii^	3.1300
C19···C31^iii^	3.525 (5)	H33C···O1	2.4000
C19···C32^iii^	3.393 (5)	H33C···N8	2.7000
C19···N1	3.405 (4)	H33C···H3	2.5700
C20···C8	3.507 (5)	H33C···H8	2.1300
C20···N1	3.424 (4)		
			
C5—O2—C17	117.6 (4)	C11—C12—H12	119.00
C22—O4—C34	120.9 (4)	C14—C13—H13	120.00
C1—N1—C8	111.3 (3)	C12—C13—H13	120.00
N3—N2—C2	119.8 (3)	C10—C15—H15	121.00
N2—N3—C9	118.9 (3)	C14—C15—H15	121.00
C9—N4—C10	130.8 (3)	C11—C16—H16A	110.00
C1—N1—H1	124.00	H16A—C16—H16C	109.00
C8—N1—H1	124.00	H16B—C16—H16C	109.00
N2—N3—H3	121.00	H16A—C16—H16B	110.00
C9—N3—H3	121.00	C11—C16—H16B	109.00
C9—N4—H4A	115.00	C11—C16—H16C	109.00
C10—N4—H4A	115.00	O3—C18—N5	128.5 (3)
C18—N5—C25	112.1 (3)	O3—C18—C19	126.3 (3)
N7—N6—C19	119.3 (2)	N5—C18—C19	105.2 (3)
N6—N7—C26	119.9 (2)	N6—C19—C18	129.8 (3)
C26—N8—C27	133.7 (3)	N6—C19—C20	123.7 (3)
C25—N5—H5	124.00	C18—C19—C20	106.5 (3)
C18—N5—H5	124.00	C19—C20—C25	106.9 (3)
C26—N7—H7A	120.00	C19—C20—C21	131.6 (3)
N6—N7—H7A	120.00	C21—C20—C25	121.6 (3)
C27—N8—H8	113.00	C20—C21—C22	116.9 (3)
C26—N8—H8	113.00	O4—C22—C21	123.7 (4)
O1—C1—C2	127.0 (3)	C21—C22—C23	122.3 (4)
N1—C1—C2	105.4 (3)	O4—C22—C23	113.8 (3)
O1—C1—N1	127.6 (3)	C22—C23—C24	121.0 (3)
N2—C2—C1	128.5 (3)	C23—C24—C25	117.1 (4)
N2—C2—C3	124.6 (3)	N5—C25—C24	129.9 (3)
C1—C2—C3	106.9 (3)	N5—C25—C20	109.1 (3)
C2—C3—C4	132.3 (3)	C20—C25—C24	121.0 (3)
C4—C3—C8	121.1 (3)	S2—C26—N8	129.8 (3)
C2—C3—C8	106.6 (3)	S2—C26—N7	118.1 (2)
C3—C4—C5	117.1 (3)	N7—C26—N8	112.1 (3)
C4—C5—C6	122.1 (3)	C28—C27—C32	121.1 (3)
O2—C5—C6	118.8 (3)	N8—C27—C28	115.8 (3)
O2—C5—C4	118.8 (3)	N8—C27—C32	123.1 (3)
C5—C6—C7	120.7 (3)	C27—C28—C33	122.1 (3)
C6—C7—C8	118.1 (3)	C29—C28—C33	121.0 (3)
N1—C8—C3	109.9 (3)	C27—C28—C29	116.9 (3)
C3—C8—C7	120.9 (3)	C28—C29—C30	123.1 (4)
N1—C8—C7	129.3 (3)	C29—C30—C31	118.8 (3)
N3—C9—N4	113.4 (3)	C30—C31—C32	121.3 (3)
S1—C9—N4	128.0 (3)	Cl2—C31—C32	118.2 (3)
S1—C9—N3	118.7 (2)	Cl2—C31—C30	120.5 (3)
N4—C10—C15	121.9 (3)	C27—C32—C31	118.8 (3)
C11—C10—C15	121.6 (3)	F6—C34—O4	111.7 (5)
N4—C10—C11	116.4 (3)	F4—C34—F5	101.4 (5)
C10—C11—C12	117.5 (4)	F4—C34—F6	107.2 (5)
C10—C11—C16	122.6 (3)	F4—C34—O4	114.5 (6)
C12—C11—C16	119.8 (4)	F5—C34—F6	107.1 (5)
C11—C12—C13	122.1 (5)	F5—C34—O4	114.1 (5)
C12—C13—C14	119.3 (5)	C20—C21—H21	122.00
Cl1—C14—C15	119.8 (3)	C22—C21—H21	121.00
C13—C14—C15	120.9 (4)	C22—C23—H23	119.00
Cl1—C14—C13	119.3 (3)	C24—C23—H23	120.00
C10—C15—C14	118.6 (4)	C23—C24—H24	121.00
F2—C17—O2	109.5 (6)	C25—C24—H24	122.00
F3—C17—O2	113.4 (6)	C28—C29—H29	118.00
F2—C17—F3	108.2 (5)	C30—C29—H29	118.00
F1—C17—F3	104.7 (6)	C29—C30—H30	121.00
F1—C17—O2	112.9 (5)	C31—C30—H30	121.00
F1—C17—F2	107.9 (5)	C27—C32—H32	121.00
C3—C4—H4	121.00	C31—C32—H32	121.00
C5—C4—H4	121.00	C28—C33—H33A	109.00
C5—C6—H6	120.00	C28—C33—H33B	110.00
C7—C6—H6	120.00	C28—C33—H33C	110.00
C6—C7—H7	121.00	H33A—C33—H33B	109.00
C8—C7—H7	121.00	H33A—C33—H33C	109.00
C13—C12—H12	119.00	H33B—C33—H33C	109.00
			
C17—O2—C5—C4	87.7 (5)	C4—C5—C6—C7	−0.3 (5)
C17—O2—C5—C6	−97.9 (5)	O2—C5—C6—C7	−174.6 (3)
C5—O2—C17—F1	59.2 (7)	C5—C6—C7—C8	0.5 (5)
C5—O2—C17—F2	179.4 (4)	C6—C7—C8—N1	178.0 (3)
C5—O2—C17—F3	−59.7 (7)	C6—C7—C8—C3	−0.8 (5)
C22—O4—C34—F4	44.9 (6)	C15—C10—C11—C12	−1.1 (5)
C34—O4—C22—C21	33.5 (7)	N4—C10—C11—C16	0.2 (5)
C34—O4—C22—C23	−150.4 (5)	C11—C10—C15—C14	1.5 (5)
C22—O4—C34—F5	−71.3 (7)	C15—C10—C11—C16	176.3 (3)
C22—O4—C34—F6	167.1 (4)	N4—C10—C15—C14	177.4 (3)
C8—N1—C1—O1	−177.9 (3)	N4—C10—C11—C12	−177.2 (3)
C8—N1—C1—C2	0.7 (3)	C10—C11—C12—C13	−0.5 (6)
C1—N1—C8—C3	−0.2 (4)	C16—C11—C12—C13	−178.0 (4)
C1—N1—C8—C7	−179.1 (3)	C11—C12—C13—C14	1.7 (7)
N3—N2—C2—C3	179.6 (3)	C12—C13—C14—Cl1	177.5 (4)
N3—N2—C2—C1	−1.0 (5)	C12—C13—C14—C15	−1.3 (7)
C2—N2—N3—C9	176.7 (3)	Cl1—C14—C15—C10	−179.1 (3)
N2—N3—C9—N4	2.9 (4)	C13—C14—C15—C10	−0.3 (6)
N2—N3—C9—S1	−176.7 (2)	O3—C18—C19—N6	−6.6 (6)
C10—N4—C9—N3	177.6 (3)	O3—C18—C19—C20	174.5 (3)
C9—N4—C10—C11	−139.6 (4)	N5—C18—C19—N6	173.9 (3)
C10—N4—C9—S1	−2.8 (6)	N5—C18—C19—C20	−5.0 (4)
C9—N4—C10—C15	44.3 (6)	N6—C19—C20—C21	5.1 (6)
C25—N5—C18—O3	−175.1 (3)	N6—C19—C20—C25	−175.2 (3)
C25—N5—C18—C19	4.4 (4)	C18—C19—C20—C21	−176.0 (3)
C18—N5—C25—C20	−2.2 (4)	C18—C19—C20—C25	3.7 (4)
C18—N5—C25—C24	177.6 (4)	C19—C20—C21—C22	179.2 (4)
C19—N6—N7—C26	−179.3 (3)	C25—C20—C21—C22	−0.5 (5)
N7—N6—C19—C20	178.4 (3)	C19—C20—C25—N5	−1.2 (4)
N7—N6—C19—C18	−0.3 (5)	C19—C20—C25—C24	179.0 (3)
N6—N7—C26—N8	−0.1 (4)	C21—C20—C25—N5	178.6 (3)
N6—N7—C26—S2	−180.0 (2)	C21—C20—C25—C24	−1.2 (5)
C27—N8—C26—S2	1.5 (6)	C20—C21—C22—O4	177.0 (4)
C27—N8—C26—N7	−178.3 (3)	C20—C21—C22—C23	1.2 (6)
C26—N8—C27—C28	−176.9 (3)	O4—C22—C23—C24	−176.6 (4)
C26—N8—C27—C32	3.9 (6)	C21—C22—C23—C24	−0.4 (7)
O1—C1—C2—N2	−1.8 (6)	C22—C23—C24—C25	−1.3 (6)
O1—C1—C2—C3	177.7 (3)	C23—C24—C25—N5	−177.7 (4)
N1—C1—C2—N2	179.6 (3)	C23—C24—C25—C20	2.0 (6)
N1—C1—C2—C3	−1.0 (3)	N8—C27—C28—C29	−178.4 (3)
C1—C2—C3—C4	178.2 (4)	N8—C27—C28—C33	1.0 (5)
C1—C2—C3—C8	0.9 (4)	C32—C27—C28—C29	0.8 (5)
N2—C2—C3—C8	−179.6 (3)	C32—C27—C28—C33	−179.8 (3)
N2—C2—C3—C4	−2.3 (6)	N8—C27—C32—C31	179.1 (3)
C2—C3—C8—N1	−0.5 (4)	C28—C27—C32—C31	−0.1 (5)
C2—C3—C4—C5	−177.6 (3)	C27—C28—C29—C30	−0.8 (5)
C8—C3—C4—C5	−0.5 (5)	C33—C28—C29—C30	179.8 (3)
C4—C3—C8—C7	0.9 (5)	C28—C29—C30—C31	0.1 (6)
C2—C3—C8—C7	178.6 (3)	C29—C30—C31—Cl2	−178.3 (3)
C4—C3—C8—N1	−178.2 (3)	C29—C30—C31—C32	0.7 (5)
C3—C4—C5—O2	174.5 (3)	Cl2—C31—C32—C27	178.3 (3)
C3—C4—C5—C6	0.3 (5)	C30—C31—C32—C27	−0.6 (5)

Symmetry codes: (i) −*x*+2, −*y*+2, −*z*; (ii) −*x*+2, −*y*+2, −*z*+1; (iii) −*x*+1, −*y*+1, −*z*+1; (iv) −*x*+1, −*y*+2, −*z*+1; (v) *x*+1, *y*+1, *z*; (vi) −*x*+1, −*y*+2, −*z*; (vii) −*x*, −*y*+2, −*z*; (viii) −*x*, −*y*+1, −*z*+1; (ix) *x*−1, *y*−1, *z*; (x) *x*−1, *y*, *z*; (xi) *x*+1, *y*, *z*.

### Hydrogen-bond geometry (Å, °)

**Table:**

*D*—H···*A*	*D*—H	H···*A*	*D*···*A*	*D*—H···*A*
N1—H1···O3^viii^	0.8600	2.1800	2.937 (3)	147.00
N3—H3···O1	0.8600	2.2100	2.841 (3)	131.00
N4—H4A···N2	0.8600	2.0900	2.553 (4)	113.00
N5—H5···S1^ix^	0.8600	2.6400	3.287 (3)	133.00
N7—H7A···O3	0.8600	2.2200	2.863 (3)	132.00
N8—H8···N6	0.8600	2.0600	2.546 (3)	115.00
C12—H12···F6^vi^	0.9300	2.4700	3.284 (6)	147.00
C15—H15···S1	0.9300	2.8700	3.325 (3)	111.00
C21—H21···F5	0.9300	2.3800	2.930 (5)	118.00
C32—H32···S2	0.9300	2.5700	3.245 (4)	130.00
C33—H33C···O1	0.9600	2.4000	3.355 (4)	176.00

Symmetry codes: (viii) −*x*, −*y*+1, −*z*+1; (ix) *x*−1, *y*−1, *z*; (vi) −*x*+1, −*y*+2, −*z*.
